# Prostate-specific antigen in serum of women with breast cancer.

**DOI:** 10.1038/bjc.1995.401

**Published:** 1995-09

**Authors:** M. Giai, H. Yu, R. Roagna, R. Ponzone, D. Katsaros, M. A. Levesque, E. P. Diamandis

**Affiliations:** Department of Gynecologic Oncology, University of Turin, Italy.

## Abstract

Prostate-specific antigen (PSA) was recently found in 30% of female breast tumours. In this study we have examined if PSA circulates in the blood of breast cancer patients and if serum PSA has any clinical application. We have compared serum PSA levels between women with and without breast cancer, between women with PSA-positive and PSA-negative breast cancer and between women with breast cancer before and after surgical removal of the tumour. We found that for women > or = 50 years, there is no difference in serum PSA between normal or breast cancer patients. We also could not find any difference in presurgical or post-surgical serum PSA between women who have PSA-positive or PSA-negative breast cancer. We found no correlation between PSA concentrations in matched presurgical and post-surgical sera, between presurgical sera and tumour cytosols and between post-surgical sera and tumour cytosols. High-performance liquid chromatography has shown that PSA in normal male serum consists mostly of PSA bound to alpha 1-antichymotrypsin (molecular weight approximately 100,000), and PSA in breast tumours and presurgical and post-surgical serum consists mostly of free PSA (molecular weight approximately 33,000). These data suggest that female serum PSA is not associated with tumour PSA levels. We speculate that most of the circulating PSA in women originates from the normal breast. It appears that serum PSA in women does not have potential for breast cancer diagnosis or monitoring, but our previous data are consistent with the view that tumour PSA concentration is a favourable prognostic indicator in women with breast cancer.


British Journal of Cancer (1995) 72, 728-731

© 1995 Stockton Press All rights reserved 0007-0920/95 $12.00

### Prostate-specific antigen in serum of women with breast cancer

M Giai[1], H Yu[2,3], R Roagna[1], R Ponzone[1], D Katsaros[1], MA Levesque[2,3] and EP Diamandis[2,3]

[1]*Department of Gynecologic Oncology, Institute of Obstetrics and Gynecology, University of Turin, Turin, Italy;* [2]*Department of Pathology and Laboratory Medicine, Mount Sinai Hospital, 600 University Avenue, Toronto, Ontario M5G 1X5, Canada;* [3]*Department of Clinical Biochemistry, University of Toronto, 100 College Street, Toronto, Ontario M5G 1L5, Canada.*

**Summary** Prostate-specific antigen (PSA) was recently found in 30% of female breast tumours. In this study we have examined if PSA circulates in the blood of breast cancer patients and if serum PSA has any clinical application. We have compared serum PSA levels between women with and without breast cancer, between women with PSA-positive and PSA-negative breast cancer and between women with breast cancer before and after surgical removal of the tumour. We found that for women ⩾ 50 years, there is no difference in serum PSA between normal or breast cancer patients. We also could not find any difference in presurgical or post-surgical serum PSA between women who have PSA-positive or PSA-negative breast cancer. We found no correlation between PSA concentrations in matched presurgical and post-surgical sera, between presurgical sera and tumour cytosols and between post-surgical sera and tumour cytosols. High-performance liquid chromatography has shown that PSA in normal male serum consists mostly of PSA bound to $\alpha_1$-antichymotrypsin (molecular weight approximately 100 000), and PSA in breast tumours and presurgical and post-surgical serum consists mostly of free PSA (molecular weight approximately 33 000). These data suggest that female serum PSA is not associated with tumour PSA levels. We speculate that most of the circulating PSA in women originates from the normal breast. It appears that serum PSA in women does not have potential for breast cancer diagnosis or monitoring, but our previous data are consistent with the view that tumour PSA concentration is a favourable prognostic indicator in women with breast cancer.

**Keywords:** prostate-specific antigen; tumour markers; steroid hormones

Prostate-specific antigen (PSA) is a 33 kDa serine protease found at high concentrations in seminal plasma and prostate epithelial cells and at relatively low concentrations in male serum (Oesterling, 1991: Armbruster, 1993). PSA production is regulated by androgenic steroids, which bind to androgen receptors and up-regulate transcription of the PSA gene (Young *et al.*, 1991). PSA was until recently thought to be produced only by prostatic epithelial cells and is currently used as a biochemical marker for diagnosis and monitoring of prostate adenocarcinoma. We have reported that PSA concentrations ⩾ 0.03 ng per mg of total protein could be detected in 30% of cytosolic extracts from female breast tumours (Yu *et al.*, 1994). The PSA immunoreactive species in female breast cancer has molecular weight identical to PSA from seminal plasma (Diamandis *et al.*, 1994). PSA mRNA was identified by polymerase chain reaction in PSA protein-positive breast tumours but not in PSA protein-negative breast tumours (Monne *et al.*, 1994). The PSA cDNA from breast tumours was identical in sequence to PSA cDNA from prostatic tissue (Monne *et al.*, 1994). Preliminary clinical studies have shown that PSA in breast cancer is associated with the presence of the progesterone receptor (Yu *et al.*, 1994) and that patients with PSA-positive tumours have a lower risk of relapse and death in comparison with patients whose tumours are PSA negative (Yu *et al.*, 1995*a*). Thus, PSA is a new candidate favourable prognostic indicator in female breast cancer.

A number of previous studies have shown that PSA is undetectable in the serum of most women (Chan *et al.*, 1987; Rock *et al.*, 1987; Vihko *et al.*, 1990) and that fewer than 5% of women have serum PSA concentrations ⩾ 0.05 $\mu g\ l^{-1}$ (Yu and Diamandis, 1995*a*). No study has as yet been published examining whether serum PSA concentrations are higher in women with breast cancer than in healthy controls or whether the PSA levels in the breast tumour affect the PSA concentration in the serum. Currently, there is no established diagnostic value of PSA measurements in female serum. This study was conducted in an attempt to answer the questions raised above and further examine if serum PSA measurements in female serum have any diagnostic, prognostic or monitoring value. We have compared serum PSA levels between women with and without breast cancer, between women with PSA-positive and PSA-negative breast cancer and between women with breast cancer before and after surgical removal of tumour.

Correspondence: EP Diamandis, Department of Pathology and Laboratory Medicine, Mount Sinai Hospital, 600 University Avenue, Toronto, Ontario M5G 1X5, Canada
Received 3 March 1995; accepted 7 April 1995

### Materials and methods

### *Human subjects*

Two hundred patients with primary breast cancer, operated on at the Department of Gynecologic Oncology, University of Turin, Italy, between January 1992 and May 1993, were included in this study. These patients represent consecutive cases for which sufficient tumour tissue remained after routine pathological examination and steroid hormone receptor analysis. The patients were aged between 29 and 93 with a median age of 57. The clinical stages (Spiessl *et al.*, 1989) of these patients were 44% stage I, 48% stage II and 8% stage III or IV. The tumour size ranged from 0.1 to 15 cm with a median of 2 cm. Of the 200 patients, 185 had their axillary lymph nodes examined at surgery; the median number of nodes examined was 15. Of the 185 patients, 99 were found to have cancer metastasis to their axillary lymph nodes. The major histological types of cancer in this group were invasive ductal (56%) and invasive lobular (16.5%). The rest included ductal *in situ* (2%), medullary (2%), papillary (4.5%), tubular (6.5%), inflammatory (3.5%), tubulo-lobular (4%), muciparous (2.5%) and others (2.5%).

Of the 200 patients, 198 had their presurgical serum collected and stored at −20°C, 199 had their cancer tissue specimens collected at surgery and stored at −70°C (snap-frozen tumour tissue) and 119 had their post-surgical serum taken 6 months after surgery and stored at −20°C.

Pre- and post-surgical sera from another 346 breast cancer patients were also collected. These patients were aged between 26 and 91 with a median age of 60. Clinical information and tumour specimens were not available for these patients.

Sera provided by the Red Cross Blood Transfusion Service in Toronto were collected from 674 female blood donors between 17 and 69 years of age with a median age of 35. These sera were taken from healthy women without clinically diagnosed breast cancer. The blood donors were considered the normal control group.

*Cytosol preparation*

Tumour tissue specimens were extracted as follows. Approximately 0.2 g of tissue from each tumour was pulverised manually with a hammer to a fine powder at −80°C. The cells were lysed for 30 min on ice with 1 ml of lysis buffer (50 mmol $l^{-1}$ Tris buffer, pH 8.0, containing 150 mmol $l^{-1}$ sodium chloride, 5 mmol $l^{-1}$ EDTA, 10 g $l^{-1}$ Nonidet NP-40 surfactant and 1 mmol $l^{-1}$ phenylmethysulphonyl fluoride). The lysates were centrifuged at 15 000 *g* at 4°C for 30 min and the supernatants (cytosolic fractions) were assayed for PSA and total protein.

*High-performance liquid chromatography (HPLC)*

In order to compare the molecular weight of PSA in female serum, breast cancer cytosol and male serum, HPLC analysis was performed with a Shimadzu system (Shimadzu, Kyoto, Japan), using a mobile phase of 0.1 mol $l^{-1}$ sodium sulphate and 0.1 mol $l^{-1}$ sodium dihydrogen phosphate, pH 6.80. The column used was a Bio-Sil SEC-250, 600 mm × 75 mm (BioRad Labs, Richmond, CA, USA) and was calibrated with a molecular weight standard solution from BioRad. The flow rate was 0.5 ml $min^{-1}$. After injection of 50–300 µl of each sample, fractions of 0.5 ml were collected and analysed for PSA using the method outlined below.

*Measurement of PSA and total protein*

PSA concentration in serum and in tumour cytosol was measured in duplicate with a time-resolved immunofluorometric PSA assay (Yu and Diamandis, 1993), which has a biological detection limit of 0.01 µg $l^{-1}$. PSA concentration in tumour cytosols was expressed as ng of PSA per mg of total protein. The total protein concentration (mg $ml^{-1}$) in the cytosols was measured in duplicate using a commercial kit based on the bicinchoninic acid method (Pierce, Rockford, IL, USA).

*Statistical analysis*

PSA concentrations in serum were categorised into three groups as follows: PSA < 0.010 µg $l^{-1}$, PSA between 0.010 and 0.029 µg $l^{-1}$ and PSA ⩾ 0.030 µg $l^{-1}$. Using the contingency table and chi-square test (or Fisher's exact test when necessary), we compared the differences in PSA concentration between breast cancer patients and normal women, between presurgical and post-surgical serum and between patients with PSA-positive and PSA-negative breast cancer. PSA-positive breast cancer was defined as a cancer with a PSA concentration ⩾ 0.03 ng $mg^{-1}$ in the tumour cytosol. The selection of this cut-off point was based on criteria described elsewhere (Diamandis *et al.*, 1994).

Pearson correlation coefficients were calculated for PSA concentrations between matched (i.e. from the same patient) presurgical and post-surgical sera, matched presurgical sera and tumour cytosols and matched post-surgical sera and tumour cytosols. The McNemar test was also used for comparison of PSA status, categorised into positive and negative groups with a cut-off level of 0.01 µg $ml^{-1}$ for serum and 0.03 ng $mg^{-1}$ for tumour cytosols.

### Results

The distribution of PSA concentrations in the serum of healthy women, the serum of women with breast cancer before or after surgery and in breast cancer cytosols is shown in Table I. The percentage of sera from presurgical breast cancer patients with PSA ⩾ 0.03 µg $l^{-1}$ is higher than the percentage of normal sera (11% *vs* 4%, $P = 0.001$). Similarly, the percentage of sera from post-surgical breast cancer patients with PSA ⩾ 0.03 µg $l^{-1}$ is higher than the percentage of normal sera (9% *vs* 4%, $P = 0.045$). No difference was seen between presurgical and post-surgical sera (11% *vs* 9%, $P = 0.86$). The percentage of breast tumour cytosols with PSA ⩾ 0.03 ng $mg^{-1}$ (26–33%) is higher than the percentage of sera of either normal or breast cancer patients ($P < 0.001$ for all comparisons and all age groups).

Similar statistical analysis was performed after all subjects were classified into two age groups, i.e. < 50 years and ⩾ 50 years. In the younger age group, the percentage of sera with PSA ⩾ 0.03 µg $l^{-1}$ was still higher in the presurgical breast cancer group than in the healthy group (12% *vs* 4%, $P = 0.003$). However, no difference was seen between sera from healthy women and women with breast cancer, post surgery (3% *vs* 4%, $P = 0.52$). In the older age group the percentages of sera with PSA ⩾ 0.03 µg $l^{-1}$ were similar in the healthy, presurgical or post-surgical groups (9% *vs* 11% *vs* 10%, $P > 0.8$ in all cases) (Table I).

We have further compared the percentages of sera with PSA ⩾ 0.03 µg $l^{-1}$ between the group of breast cancer patients without any clinical information ($n = 346$) and healthy women. No statistically significant difference was found ($P = 0.093$ for ages < 50 years and $P = 0.33$ for ages ⩾ 50 years).

The distribution of PSA concentrations in the serum of presurgical or post-surgical breast cancer patients whose tumours are either PSA positive or PSA negative is shown in Table II. The percentage of presurgical sera with PSA ⩾ 0.03 µg $l^{-1}$ was higher when the tumours were PSA negative, but the difference was not statistically significant (13% *vs* 7%, $P = 0.053$). Similarly, no difference was seen

**Table I** PSA concentration in the serum of healthy women, breast cancer patients before and after surgery and in breast tumour cytosols

| Sample | PSA (µg $l^{-1}$ in serum or ng $mg^{-1}$ in cytosol) < 0.010 | 0.010–0.029 | ⩾ 0.030 | Total |
|---|---|---|---|---|
| 1. Serum of healthy women[a] | | | | |
| All subjects | 561 (83.2)[b] | 86 (12.8) | 27 (4.0) | 674 |
| < 50 years | 478 (83.6) | 76 (13.3) | 18 (3.1) | 572 |
| ⩾ 50 years | 83 (81.4) | 10 (9.8) | 9 (8.8) | 102 |
| 2. Serum of breast cancer patients before surgery | | | | |
| All subjects | 154 (77.8) | 22 (11.1) | 22 (11.1) | 198 |
| < 50 years | 44 (77.2) | 6 (10.5) | 7 (12.3) | 57 |
| ⩾ 50 years | 110 (78.0) | 16 (11.4) | 15 (10.6) | 141 |
| 3. Serum of breast cancer patients post surgery | | | | |
| All subjects | 95 (79.8) | 13 (10.9) | 11 (9.3) | 119 |
| < 50 years | 18 (78.3) | 4 (17.4) | 1 (4.3) | 23 |
| ⩾ 50 years | 77 (80.2) | 9 (9.4) | 10 (10.4) | 96 |
| 4. Tumour cytosolic extracts | | | | |
| All patients | 40 (20.1) | 103 (51.8) | 56 (28.1) | 199 |
| < 50 years | 9 (15.8) | 29 (50.9) | 19 (33.3) | 57 |
| ⩾ 50 years | 31 (21.8) | 74 (52.1) | 37 (26.1) | 142 |
| 5. Serum of breast cancer patients[c] | | | | |
| All patients | 299 (86.4) | 23 (6.7) | 24 (6.9) | 346 |
| < 50 years | 67 (83.8) | 7 (8.7) | 6 (7.5) | 80 |
| ⩾ 50 years | 232 (87.2) | 16 (6.0) | 18 (6.8) | 266 |

[a]Statistical analysis – all subjects, two degrees of freedom: $\chi^2 = 14.6$ $P = 0.001$, for 1 and 2; $\chi^2 = 6.2$, $P = 0.045$, for 1 and 3; $\chi^2 = 0.29$, $P = 0.86$, for 2 and 3. Patients < 50 years, two degrees of freedom: $\chi^2 = 11.4$, $P = 0.003$, for 1 and 2; $P = 0.52$ with Fisher's exact test (two-tail) for 1 and 3; $P = 0.47$ with Fisher's exact test for 2 and 3. Patients ⩾ 50 years: $\chi^2 = 0.41$, $P = 0.81$, for 1 and 2; $\chi^2 = 0.15$, $P = 0.93$, for 1 and 3; $\chi^2 = 0.25$, $P = 0.88$, for 2 and 3. Other data are presented in the text. [b]Number of patients with percentage in brackets. [c]For this group of breast cancer patients no clinical information was available.

between the sera from post-surgical patients with PSA-positive or PSA-negative tumours ($P = 0.45$, Table II).

We have also correlated the PSA concentrations between presurgical and post-surgical sera ($n = 118$ pairs), between presurgical sera and tumour cytosols ($n = 197$) and between post-surgical sera and tumour cytosols ($n = 119$). The Pearson correlation coefficients were all below 0.02 and none was statistically significant ($P \geqslant 0.78$ in all three cases.

We have also analysed the possible associations of PSA levels between presurgical sera and tumour cytosols using categorical data and the McNemar test. PSA cut-off levels used were 0.01 $\mu g\ l^{-1}$ for serum and 0.03 ng $mg^{-1}$ for the tumours. There were 109 serum/tumour pairs negative for PSA, 33 pairs which were positive for PSA in the serum and negative in the tumour, 44 pairs which were negative in the serum and positive in the tumour and 11 pairs positive for PSA in both the serum and tumour. The $P$-value was 0.21, indicating no statistical significance.

From the statistical analysis we concluded that high levels of PSA in the tumour were not correlated with high PSA levels in the presurgical sera.

We have performed high-performance liquid chromatographic separation of the serum PSA subfractions (Figure 1). For this experiment we used a serum from a normal male, a PSA-positive breast tumour cytosol, a serum from a female with breast cancer collected before surgery and a serum from a female with breast cancer collected post surgery. Normal male serum contains both free PSA (PSA) and PSA bound to $\alpha_1$-antichymotrypsin (ACT-PSA), the predominant form being ACT-PSA. In breast tumours and female serum the major fraction appears to be free PSA.

### Discussion

As PSA is found in 30% of breast cancer cytosols, it is worthwhile examining if PSA is also present in the serum of breast cancer patients and if the serum levels have any clinical implication. We were able to study this question in detail by simultaneously examining tumour PSA levels and matched presurgical and post-surgical serum samples as well as sera from normal women.

Comparisons of serum PSA levels between 674 normal women, 198 women with breast cancer from Italy and 346

**Table II** PSA concentration in presurgical and post-surgical sera from patients with either PSA-positive or PSA-negative breast cancer

| | *PSA ($\mu g\ l^{-1}$)* | | | |
|---|---|---|---|---|
| | *<0.01* | *0.01–0.029* | *≥0.030* | *P* |
| *Presurgical serum* | | | | |
| PSA-positive cancer | 44 (80%) | 7 (13%) | 4 (7%) | |
| PSA-negative cancer | 109 (77%) | 15 (11%) | 18 (13%) | 0.53[a] |
| *Post-surgical serum* | | | | |
| PSA-positive cancer | 32 (87%) | 2 (5%) | 3 (8%) | |
| PSA-negative cancer | 63 (77%) | 11 (13%) | 8 (10%) | 0.45[b] |

[a]Chi-square test with two degrees of freedom. [b]Fisher's exact test (two-tail).

**Figure 1** Separation of serum PSA by high-performance liquid chromatography and assay of the fractions by the time-resolved immunofluorometric procedure. (**a**) Male serum. (**b**) Breast tumour cytosolic extract. (**c**) Presurgical female serum. (**d**) Post-surgical female serum. The PSA–$\alpha_1$-antichymotrypsin complex (ACT-PSA) elutes at fraction 30 ± 1 (molecular weight approximately 100 000). Free PSA (PSA) elutes at fraction 39 ± 1 (molecular weight approximately 33 000). The major peak in male serum is ACT-PSA. The major peak in female serum and breast tumour extracts is free PSA.

women with breast cancer from Canada have shown slight increases in PSA levels among women with breast cancer (Table I). However, since elderly normal women tend to have higher PSA levels in their serum than younger women (Yu and Diamandis, 1995*a*) and younger breast cancer patients tend to have more frequently PSA-positive breast cancer (Yu *et al*., 1994), we have further analysed the data after stratifying women according to age, i.e $<50$ years and $\geqslant 50$ years. PSA levels in sera from normal women and breast cancer patients were not different in the older patient groups. In the younger patient groups the PSA levels were slightly higher in the presurgical sera, but not in the post-surgical sera, in comparison with sera from normal women. Based on these observations it seems unlikely that the PSA levels in the serum of breast cancer patients are significantly different from the PSA levels in the serum of normal women, especially for women $>50$ years, in whom breast cancer is more prevalent.

The PSA concentration in the tumour cytosols does not seem to influence the PSA concentration in the serum of breast cancer patients. This suggestion is based on the following data. First, we did not observe higher serum PSA levels in PSA-positive cancers than in PSA-negative cancers (Table II). Second, no correlation was found between PSA levels in the presurgical sera and cancer cytosols. Third, no association was found between positive tumour and positive presurgical sera when the data were examined categorically by the McNemar test. Fourth, by examining matched presurgical and post-surgical sera, the PSA status in the serum did not change significantly when the cancer was removed.

In this study we did not find any association between serum PSA and tumour cytosol PSA. However, our previous study, which examined levels of PSA in amniotic fluid and maternal serum at various gestational ages, showed that the two values change in parallel, following a very similar pattern. Moreover, we have demonstrated that serum levels of PSA are higher in pregnant women than in non-pregnant women (Yu and Diamandis, 1995*b*). PSA was also found in the milk of lactating women and in normal breast, especially after stimulation by oral contraceptives. These two findings suggest that PSA is produced not only by breast tumours, but also by the normal breast cells (Yu and Diamandis, 1995*c*; Yu *et al*., 1995*b*).

There are several possibilities which may have obscured an association between serum PSA and tumour cytosol PSA. For example, patients who have PSA-positive cancer and PSA-negative serum may have some PSA in the serum that is not measurable by the method used. PSA levels in breast tumours are much lower than PSA levels in the prostate. Based on the ratio of PSA concentration in seminal plasma and serum (approximately $10^6$) we may expect that serum PSA originating from the breast tumour is at a level below 0.01 $\mu g\, l^{-1}$ in most cases. In the prostate, PSA enters the circulation through physical diffusion (Oesterling *et al*., 1988; Oesterling, 1991). Factors that affect the transport of PSA from the tissue to the blood may also be considered when one examines the relationship between PSA levels in the serum and tissue.

For those patients who had PSA-negative tumours and PSA-positive serum, there are at least two possible reasons to explain the phenomenon. First, it is conceivable that PSA is produced by foci of cells scattered throughout the tumour and that the examined tissue was negative owing to sampling bias. Second, we found that PSA can be produced by the normal breast tissue as well (Yu and Diamandis, 1995*c*; Yu *et al*., 1995*b*). Consequently, the serum PSA circulating in some women with or without cancer is probably released by the breast epithelial cells under stimulation by endogenous or exogenous steroid hormones. In the limited number of samples analysed, it appears that most of the PSA is present in its free form in the female serum but is present as ACT-PSA in the male serum (Figure 1). Free PSA also predominates in the breast tumour.

In summary, we found that there is no substantial difference between serum PSA levels from normal women and from women with breast cancer. No association was found between tumour PSA levels and serum PSA or between presurgical and post-surgical serum PSA levels. Based on these data we conclude that serum PSA levels are not useful for breast cancer patient diagnosis or monitoring. However, tumour levels of PSA appear to be valuable for breast cancer patient prognosis, since patients with PSA-positive tumours have much longer disease-free and overall survival (Yu *et al*., 1995*a*).

**Acknowledgements**

This work was supported by a grant to EP Diamandis from The Ontario Chapter of the Canadian Breast Cancer Foundation. Dr D Katsaros is supported by an AIRC (Associazione Italiana per la Ricerca sul Cancro) fellowship.

**References**

ARMBRUSTER DA. (1993). Prostate specific antigen: biochemistry analytical methods, and clinical application. *Clin. Chem*., **39**, 181-195.

CHAN DW, BRUZEK DJ, OESTERLING JE, ROCK RC AND WALSH PC. (1987). Prostate-specific antigen as a marker for prostate cancer: a monoclonal and a polyclonal immunoassay compared. *Clin. Chem*., **33**, 1916-1920.

DIAMANDIS EP, YU H AND SUTHERLAND DJA. (1994). Detection of prostate specific antigen immunoreactivity in breast tumours. *Breast Cancer Res. Treat*., **32**, 301-310.

MONNE M, CROCE CM, YU H AND DIAMANDIS EP. (1994). Molecular characterization of prostate specific antigen RNA expressed in breast tumor. *Cancer Res*., **54**, 6344-6347.

OESTERLING JE. (1991). Prostate specific antigen: a critical assessment of the most useful tumor marker for adenocarcinoma of the prostate. *J. Urol*., **145**, 907-923.

OESTERLING JE, CHAN DW AND EPSTEIN JI. (1988). Prostate specific antigen in the preoperative and postoperative evaluation of localized prostatic cancer treated with radical prostatectomy. *J. Urol*., **139**, 766-772.

ROCK RC, CHAN DW, BRUZEK D, WALDRON C, OESTERLING J AND WALSH PC. (1987). Evaluation of a monoclonal immunoradiometric assay for prostate-specific antigen. *Clin. Chem*., **33**, 2257-2261.

SPIESSL B, BEAHRS OH, HERMANEK P, HUTTER RVP, SCHEIBE O, SOBIN LH AND WAGNER G. (1989). *TNM Atlas. Illustrated Guide to the TNM pTNM Classification of Malignant Tumors*. Springer: New York.

VIHKO P, KURKELA R, RAMBERG J, PELKONEN I AND VIHKO R. (1990). Time-resolved immunofluorometric assay of human prostate-specific antigen. *Clin. Chem*., **26**, 92-95.

YOUNG CYF, MONTGOMERY BT, ANDREWS PE, QUI S, BILHARTZ DL AND TINDALL DJ. (1991). Hormonal regulation of prostate-specific antigen mRNA in a human prostate adenocarcinoma cell line LNCaP. *Cancer Res*., **51**, 3748-3752.

YU H AND DIAMANDIS EP. (1993). Ultrasensitive time resolved immunofluorometric assay of prostate specific antigen in serum and preliminary clinical studies. *Clin. Chem*., **39**, 2108-2114.

YU H, DIAMANDIS EP AND SUTHERLAND DJA. (1994). Immunoreactive prostate specific antigen levels in female and male breast tumors and its association with steroid hormone receptors and patient age. *Clin. Biochem*., **27**, 75-79.

YU H AND DIAMANDIS EP. (1995*a*). Measurement of serum prostate specific antigen levels in females and in prostatectomized males with an ultrasensitive immunoassay technique. *J. Urol*., **153**, 1004-1008.

YU H AND DIAMANDIS EP. (1995*b*). Prostate specific antigen immunoreactivity in amniotic fluid. *Clin. Chem*., **41**, 204-210.

YU H AND DIAMANDIS EP. (1995*c*). Prostate specific antigen is present in the milk of lactating women. *Clin. Chem*., **41**, 54-58.

YU H, GIAI M, DIAMANDIS EP, KATSAROS D, SUTHERLAND DJA, LEVESQUE MA, ROAGNA R, PONZONE R AND SISMONDI P. (1995*a*). Prostate specific antigen is a new favourable prognostic indicator for women with breast cancer. *Cancer Res*., **55**, 2104-2110.

YU H, DIAMANDIS EP, MONNE M AND CROCE CM. (1995*b*). Oral contraceptive-induced expression of prostate specific antigen in the female breast. *J. Biol. Chem*., **270**, 6615-6618.


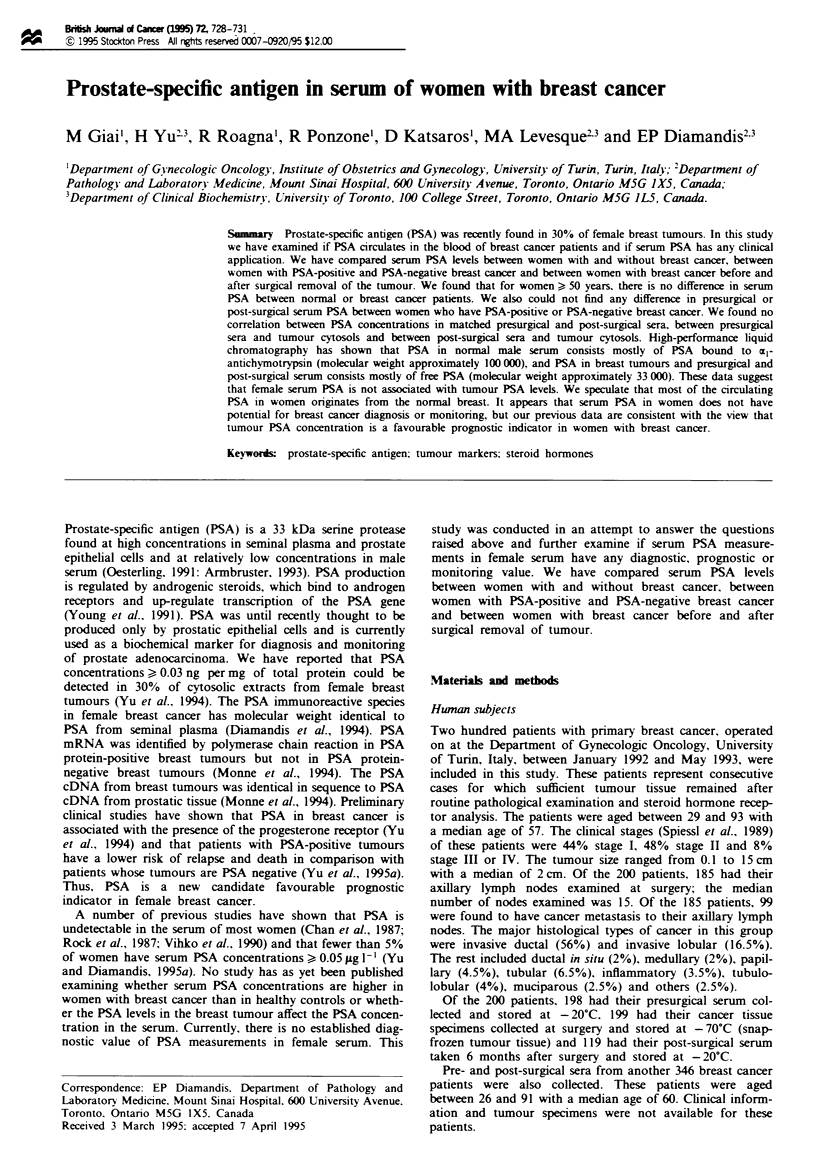

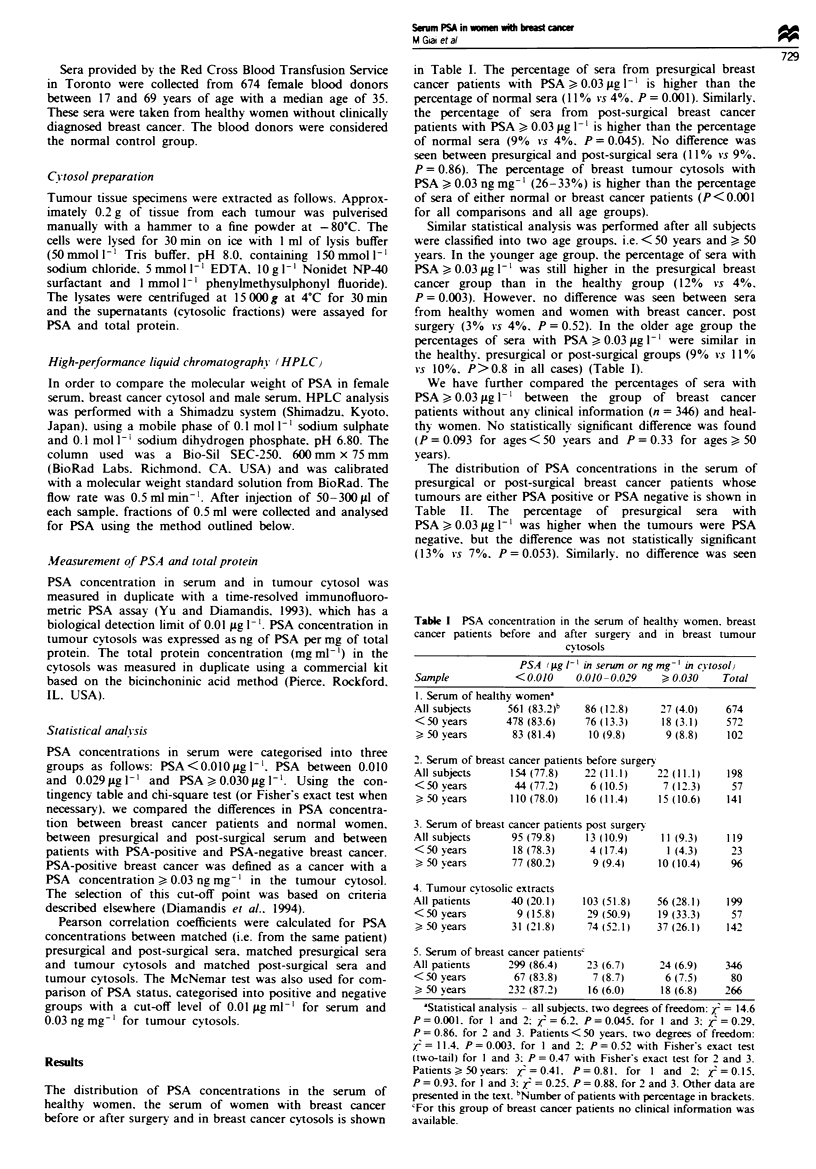

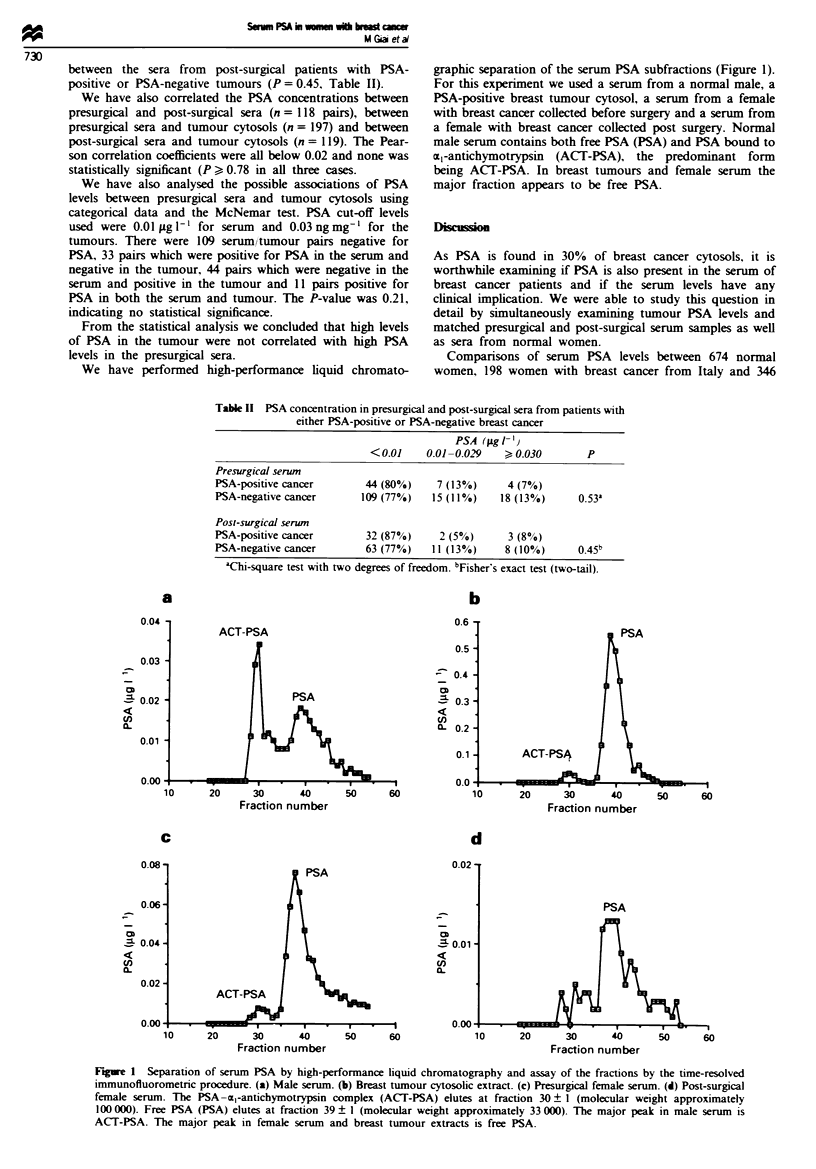

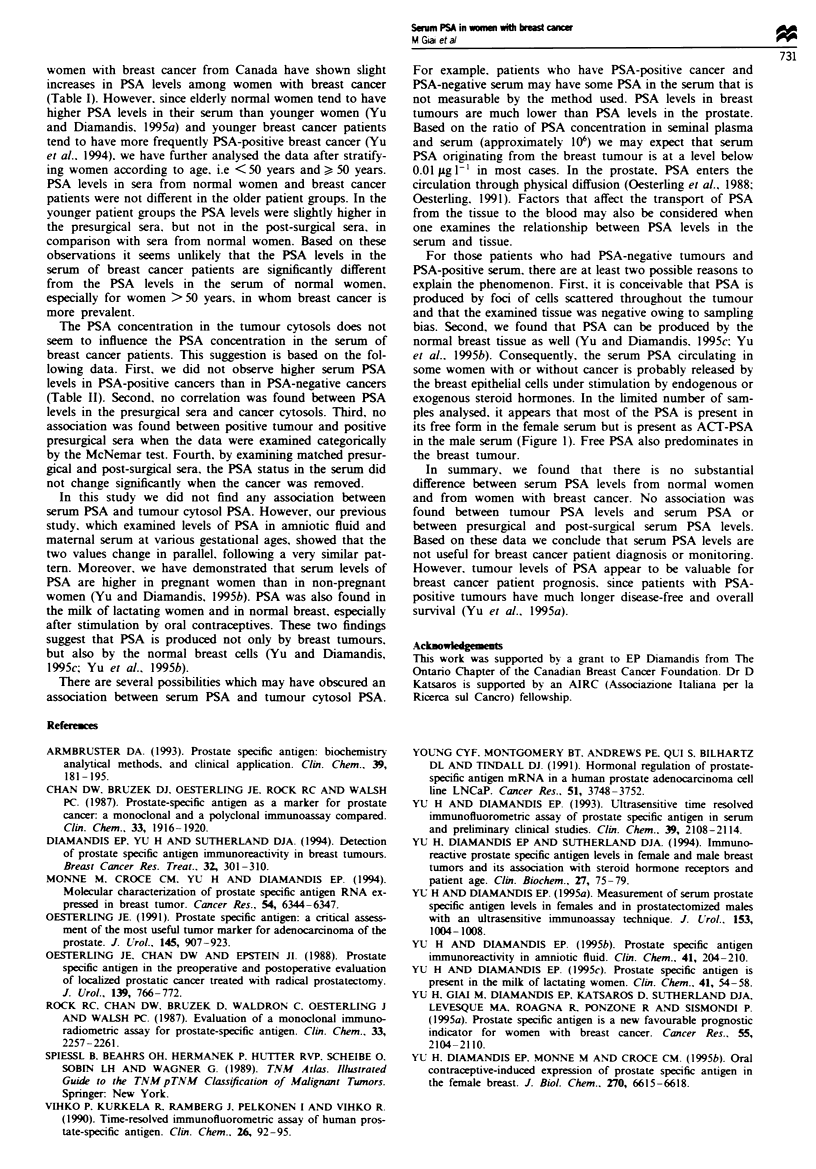


## References

[OCR_00555] Armbruster D. A. (1993). Prostate-specific antigen: biochemistry, analytical methods, and clinical application.. Clin Chem.

[OCR_00560] Chan D. W., Bruzek D. J., Oesterling J. E., Rock R. C., Walsh P. C. (1987). Prostate-specific antigen as a marker for prostatic cancer: a monoclonal and a polyclonal immunoassay compared.. Clin Chem.

[OCR_00568] Diamandis E. P., Yu H., Sutherland D. J. (1994). Detection of prostate-specific antigen immunoreactivity in breast tumors.. Breast Cancer Res Treat.

[OCR_00571] Monne M., Croce C. M., Yu H., Diamandis E. P. (1994). Molecular characterization of prostate-specific antigen messenger RNA expressed in breast tumors.. Cancer Res.

[OCR_00583] Oesterling J. E., Chan D. W., Epstein J. I., Kimball A. W., Bruzek D. J., Rock R. C., Brendler C. B., Walsh P. C. (1988). Prostate specific antigen in the preoperative and postoperative evaluation of localized prostatic cancer treated with radical prostatectomy.. J Urol.

[OCR_00578] Oesterling J. E. (1991). Prostate specific antigen: a critical assessment of the most useful tumor marker for adenocarcinoma of the prostate.. J Urol.

[OCR_00590] Rock R. C., Chan D. W., Bruzek D., Waldron C., Oesterling J., Walsh P. (1987). Evaluation of a monoclonal immunoradiometric assay for prostate-specific antigen.. Clin Chem.

[OCR_00599] Vihko P., Kurkela R., Ramberg J., Pelkonen I., Vihko R. (1990). Time-resolved immunofluorometric assay of human prostate-specific antigen.. Clin Chem.

[OCR_00607] Young C. Y., Montgomery B. T., Andrews P. E., Qui S. D., Bilhartz D. L., Tindall D. J. (1991). Hormonal regulation of prostate-specific antigen messenger RNA in human prostatic adenocarcinoma cell line LNCaP.. Cancer Res.

[OCR_00623] Yu H., Diamandis E. P. (1995). Measurement of serum prostate specific antigen levels in women and in prostatectomized men with an ultrasensitive immunoassay technique.. J Urol.

[OCR_00641] Yu H., Diamandis E. P., Monne M., Croce C. M. (1995). Oral contraceptive-induced expression of prostate-specific antigen in the female breast.. J Biol Chem.

[OCR_00627] Yu H., Diamandis E. P. (1995). Prostate-specific antigen immunoreactivity in amniotic fluid.. Clin Chem.

[OCR_00631] Yu H., Diamandis E. P. (1995). Prostate-specific antigen in milk of lactating women.. Clin Chem.

[OCR_00615] Yu H., Diamandis E. P., Sutherland D. J. (1994). Immunoreactive prostate-specific antigen levels in female and male breast tumors and its association with steroid hormone receptors and patient age.. Clin Biochem.

[OCR_00612] Yu H., Diamandis E. P. (1993). Ultrasensitive time-resolved immunofluorometric assay of prostate-specific antigen in serum and preliminary clinical studies.. Clin Chem.

[OCR_00634] Yu H., Giai M., Diamandis E. P., Katsaros D., Sutherland D. J., Levesque M. A., Roagna R., Ponzone R., Sismondi P. (1995). Prostate-specific antigen is a new favorable prognostic indicator for women with breast cancer.. Cancer Res.

